# Dataset on the influence of software development agility on software firms' performance in Bangladesh

**DOI:** 10.1016/j.dib.2019.103741

**Published:** 2019-03-07

**Authors:** Farzana Sadia, Imran Mahmud, Eva Dhar, Nusrat Jahan, Syeda Sumbul Hossain, A.K.M. Zaidi Satter

**Affiliations:** a Department of Software Engineering, Daffodil International University, Bangladesh; b Graduate School of Business, Universiti Sains Malaysia, Malaysia; c Department of Computer Science and Engineering, Daffodil International University, Bangladesh

## Abstract

The article identifies the relationship among different agile software development approaches such as response extensiveness, response efficiency, team autonomy, team diversity, and software functionality that software teams face difficult challenges in associating and achieving the right balance between the two agility dimensions. This research strategy, in terms of quantity, is descriptive and correlational. Statistical analysis of the data was carried out, using SmartPLS 3.0. Statistical population, consist of employees of software industries in Bangladesh, who were engaged in 2017 and their total number is about 100 people. The data show that the response extensiveness, response efficiency, team autonomy, team diversity, and software functionality have impact on software development agility and software development performance.

**Table:** Specifications table

Subject area	Software Engineering
More specific subject area	Software development agility and software development performance.
Type of data	Table, figure
How data was acquired	Questionnaire analysis was adopted. SmartPLS 3.0 was used to develop the model.
Data format	Raw, analyze, descriptive, statistical
Experimental factors	Agile software development approaches, which affirm sense-and-respond, self-organization and cross-functional teams were considered to determine the software development agility.
Experimental features	The relationship among response extensiveness, response efficiency, team autonomy, team diversity, and software functionality were determined
Data source location	Dhaka, Bangladesh.
Data accessibility	Data is with this article
Related research article	Lee, G., & Xia, W. (2010). Toward agile: an integrated analysis of quantitative and qualitative field data on software development agility. *Mis Quarterly*, *34*(1), 87–114.

**Table:** 

**Value of the data** • These data describe demographic data in employer of software industry of Bangladesh and the practices of agile development principles. • The dataset showed that Software team autonomy significantly influences Software Team Response Efficiency and Software team diversity significantly influences Software Team Response Extensiveness. • These data can be used to improve the factors of agile practices and increase software development performance in the software industry in Bangladesh.

## 1 Data

The dataset of this article provides the information on the recent agile software development approaches. Table 1 shows the demographic details of employers of software companies.

**Table 1:** Demographic characteristic of employers of software companies.

Parameter	Characteristics	Number (Percentage)
Organizations	Software Development &Health	57.4
	Software Development	25
	Banking/Finance/Insurance	2.9
	Consulting	5.9
	Telecommunications	7.4
	Government	1.5
	
Respondents	Quality Assurance	4.4
	Technical Project Manager	2.9
	Lead Test Engineer	1.5
	Software Engineer	1.5
	Sr. Software Engineer (android)	10.3
	Sr. Software Engineer	8.8
	Lead Software Engineer	7.4
	Senior Software Engineer (media)	8.8
	Head of Design	1.5
	Technology Lead	5.9
	Project Coordinator	1.5
	System Analyst	1.5
	Technical Writer	5.9
	Software Writer	5.9
	Software Developer	1.5
	Graphics Designer	2.9
	Senior Developer	2.9
	Developer	13.2
	Junior Developer	2.9
	Tester	1.5
Work Experience	>6	13.2
	>1	17.6
	4–6	32.4
	1–3	36.8
Company Size	<5	7.4
	21–50	17.6
	51–120	75
Budget	<10000	13.6
	10000–50000	0
	50000–100000	22.7
	100000–500000	59.1
	>500000	4.5
Project Duration	<3 months	26.5
	3–5 months	45.6
	>5 months	26.5

## 2 Experimental design, materials, and methods

20 software firms were chosen from Dhaka, Bangladesh. 160 questionnaires were distributed and 100 usable questionnaires were returned for analysis. In this study, data were gathered from all kind of software firms (small, medium, large) as well as a questionnaire [1] including the demographic data (e.g. qualification, experience). Then, the collected data were collected, coded and entered into SmartPLs 3.0. Data analysis was performed, using SPSS-21. Data were analyzed; applying descriptive and statistical tests including partial least squares approach.

### 2.1 Measurement model

Table 2 shows that composite reliability and the AVE of all variables are higher than 0.7 and 0.5 [2], [3], [4] respectively, we can state that both criterion accept our five variables.

**Table 2:** Composite Reliability and Average Variance Extracted (AVE) of variables.

	Composite Reliability	AVE
Software team autonomy (AUT)	0.901	0.695
Software team diversity (DIV)	0.908	0.712
Software Team Response Efficiency (EFI)	0.907	0.625
Software Team Response Extensiveness (EXT)	0.742	0.608
Software functionality(FUN)	0.869	0.625

Table 3 shows that the square root of the average variance where all the values on the diagonals are greater than the corresponding row and columns. It indicates that the measures are discriminant.

**Table 3:** Square root of the average variance.

	AUT	DIV	EFI	EXT	FUN
AUT	**0.834**				
DIV	0.476	**0.844**			
EFI	−0.254	−0.084	**0.790**		
EXT	0.150	0.277	0.096	**0.780**	
FUN	0.170	0.303	−0.211	0.554	**0.791**

Bold indicates to highlight that diagonal values are higher than other values.

### 2.2 Structural model

**Fig. 1:**
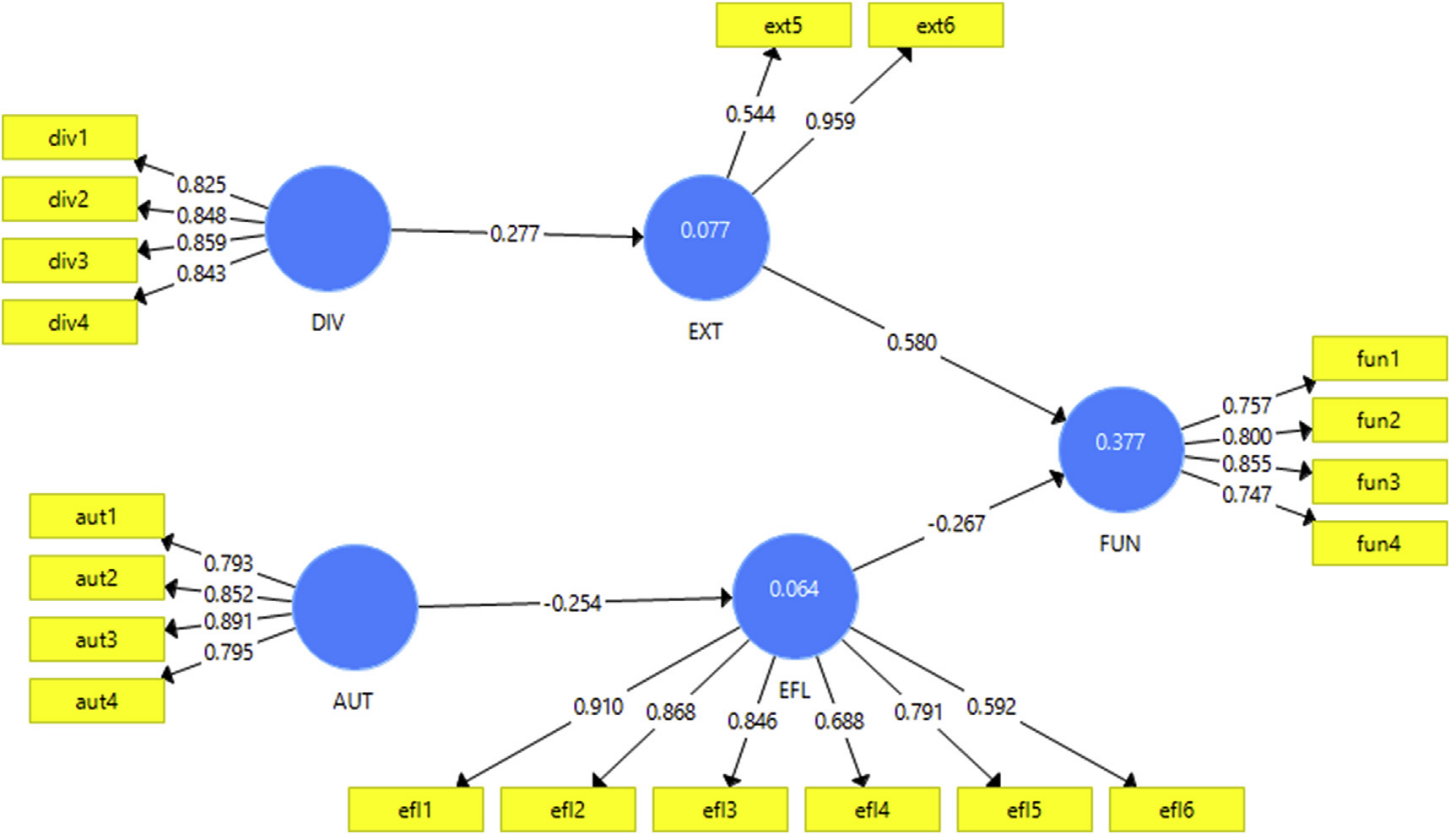
Pictorial representation of Table 4.

Table 4 presents that in the structural model the significance of the relations among variables is measured by the path coefficient. We found that Software team autonomy (AUT) (β = −0.254 and p < 0.05) significantly influences Software Team Response Efficiency (EFI), Software team diversity (DIV) (β = 0.277 and p < 0.1) significantly influences Software Team Response Extensiveness (EXT). The relationship between EFI (β = −0.267 and p < 0.05) and EXT (β = 0.580 and p < 0.05) also have significantly influence on Software functionality (FUN) (see Fig. 1).

**Table 4:** Path coefficient of the variables.

	Original Sample (O)	T Statistics (|O/STDEV|)	P Values	Result
AUT → EFI	**−0.254**	**1.737**	**0.083**	Supported*
DIV → EXT	0.277	2.224	**0.027**	Supported**
EFI → FUN	−0.267	1.827	0.068	Supported*
EXT → FUN	**0.580**	**6.748**	**0.000**	Supported**

Note: PLS estimation results (n = 100, **p < 0.05, *p < 0.1).

Bold indicates to highlight the strongly supported results.

The effect was calculated by following Cohen's effect size estimation [5]. Effect size is considered as small, medium and large if the values are 0.02, 0.15 and 0.35 respectively. Next this study also assessed effect sizes (f^2^). Besides the path coefficient also the effect size can be evaluated to control for the respective impact of different variables in one model. In our case, Table 5 shows that AUT and DIV have small effect on EFI and EXT. For the dependent variable FUN, EFI has small effect comparatively to EXT.

**Table 5:** Effect size.

	Effect Size	Remark
AUT → EFI	0.069	Small
DIV → EXT	0.083	Small
EFI → FUN	0.114	Small
EXT → FUN	0.535	Large

## Funding sources

There was no funding source for this work.
